# Layered Social Network Analysis Reveals Complex Relationships in Kindergarteners

**DOI:** 10.3389/fpsyg.2016.00276

**Published:** 2016-03-02

**Authors:** Mireille Golemiec, Jonathan Schneider, W. Thomas Boyce, Nicole R. Bush, Nancy Adler, Joel D. Levine

**Affiliations:** ^1^Department of Biology, University of Toronto MississaugaMississauga, ON, Canada; ^2^Departments of Psychiatry and Pediatrics, University of California, San FranciscoSan Francisco, CA, USA

**Keywords:** hierarchy, social, kindergarten children, social structure, layered networks, motifs

## Abstract

The interplay between individuals forms building blocks for social structure. Here, we examine the structure of behavioral interactions among kindergarten classroom with a hierarchy-neutral approach to examine all possible underlying patterns in the formation of layered networks of “reciprocal” interactions. To understand how these layers are coordinated, we used a layered motif approach. Our dual layered motif analysis can therefore be thought of as the dynamics of smaller groups that tile to create the group structure, or alternatively they provide information on what the average child would do in a given local social environment. When we examine the regulated motifs in layered networks, we find that transitivity is at least partially involved in the formation of these layered network structures. We also found complex combinations of the expected reciprocal interactions. The mechanisms used to understand social networks of kindergarten children here are also applicable on a more general scale to any group of individuals where interactions and identities can be readily observed and scored.

## Introduction

The interplay between individuals forms building blocks for social structure. In some cases such as baboon troupes and human military units, rank is evident to outside observers and within the group (Hausfater, 1974; Dean et al., 1975). In other cases, such as schools of fish (Whiteman and Cote, 2004), flocks of birds (Noble, 1939) and groups of *Drosophila* (Yurkovic et al., 2006) the presence of hierarchical relations are less evident within the group, although they may emerge in the context of resource scarcity. One method assumed to reveal hierarchy is through the evaluation of aggressive conflicts. Winners and losers throughout the animal kingdom have to deal with the consequences of battling and even when there are wounds to lick on both sides, there often appears to be a strong correlation between winners and dominance on the one hand, losers and submission on the other. There is a noteworthy caveat to this view; although dominant males may be able to guarantee access to resources on demand, others may use alternative strategies to gain access. Consider the sneaky copulator (or other alternative mating tactics Gross, 1996), for example. This “gray” area extends through all interactions between organisms. Even when the fight may be consistently “won” by a specific individual the “dominant” label is often thought to transcend aggression and imply dominance and resource monopoly (Drews, 1993). But this supposition is almost never empirically tested.

The dominance/submissive relationship is an attractive framework within which to study social interactions as it allows quantification of often complex interactions, and is known to generate hierarchies which have been proven to affect many aspects of the social organization and subsequent interactions within the group (Barroso et al., 2000; Whiteman and Cote, 2004; Sapolsky, 2005). Charting interaction patterns within these groups reveals a stratification within a group, where individuals that are socially dominant hold positions that rank higher than those who are socially subordinate. Classically, hierarchies are described as a ladder-like relationship between an alpha individual and individuals of ever-decreasing ranks where the number of rungs, and the number of individuals occupying each rung, varies (Drews, 1993). There are specific measures used to describe and understand hierarchical arrangements—stability (how consistent the arrangement is over time), steepness (ease of movement between levels, de Vries et al., 2006) and linearity or transitivity (such that if A>B and B>C then A>C, de Vries, 1995).

These measures all quantify the level of hierarchy, but stability and steepness do not have clear null hypotheses—and hierarchical and non-hierarchical categories will rely on extensive study of interactions to determine what is a biologically relevant amount of stability and steepness (e.g., not a hierarchy that flips every 5 min in primates). Transitivity on the other hand makes definite predictions about the relationships between individuals, and provides an intuitive classifier for hierarchy. Herein we examine transitivity and not hierarchy, and we posit that highly structured groups do not necessarily mean highly hierarchical groups. While any dyadic interaction may be classified as having a quantifiable “top” and “bottom,” these may simply be “roles” within a highly structured, but cooperative group, where dominant and subordinate labels are not static and dependent on the immediate social surrounding (i.e., A above B, but in the presence of C, B above A).

Many studies have shown that people innately behave in more dominant or subordinate ways when interacting, and that the roles a person takes affects education level, family background, income, and socioeconomic status (Boyce et al., 2012). In turn, these qualities also influence behaviors, leading to the seemingly-stratified system of organization we see in many human populations. Whether or not this social stratification is based on innate hierarchical relationships between interacting individuals, its effects on health and development are numerous and often begin early in childhood (Adler et al., 1994; Boyce et al., 2012). That these relationships exist and are relevant in young children opens up the possibility of studying human interactions in populations that may not be completely affected by cultural stratification but based largely on behavior. Kindergarten classrooms are therefore an excellent system within which to study the patterns of social interactions and the networks that they form.

In 2012 Boyce et al. examined the influence of socioeconomic status (SES) on the types of behavioral interactions and hierarchical positioning of kindergarten children within classrooms in Western California, suggesting that classrooms are stratified by behavioral rankings (Boyce et al., 2012). Here we use the same data set (described briefly in methods and in detail in Boyce et al., 2012) to examine all interactions within classrooms settings. We focus on six main interaction types, forming three reciprocal pairings (Resource Struggle and Prosocial, Aggression and Submission, and Leadership and Followship), removing hierarchy-specific quantitative classifications of individual children made by the observers. While the first four interaction types are easily understood, resource struggle and prosocial are less intuitive. We follow Boyce's definition of prosocial behavior as “a voluntary behavior to benefit another child” and resource struggle as the opposite, where a child struggles over access to either an object or the attention of another student or teacher (Boyce et al., 2012). These interactions are often considered reciprocal such that outputting one ensures input of the other (i.e., being led by someone equates to following them, struggling for a resource once may establish a dynamic of resource flow between individuals). Furthermore, our data set does not include emotional reactions to the physical/verbal interactions being observed and as such, bullying-type behaviors were not examined.

We first look at children's interactions through time from the vantage point of the average child. We use transition matrices to examine probabilities of moving from one interaction type to another. Using principal component analysis on these transition matrices allows us to look for group separation in the students based on their likelihood of interacting in a specific order. While this technique illustrates the effects of previous interactions (incoming or outgoing) on the average child's next interaction, a child's behavior is not only dependent on their past social experiences but also where they are situated in their social environment. To understand a kindergartener's interactions in the context of their broader social relationships, we use social network analysis.

While network analysis typically relies on a single network, our analysis applies an average across multiple samples to determine differences (Schneider et al., 2012; Kim et al., 2015). We use standardized *Z*-scores which allow us to ask about the regulation above or below what is expected of a null model (randomizing who is connected to who while maintaining the distribution of individual interactions). Up regulation (significantly positive *Z*-scores) implies active behavioral mechanisms are increasing the prevalence of a given motif. Conversely down regulation (significantly negative *Z*-scores) implies that behavioral mechanisms avoid the patterns of given motifs. Additionally we extended our network analysis to work with two behavioral interactions (one layered on top of the other). These multilayer networks often inform on the network dynamics (including robustness and transmission speed; see (Kivelä et al., 2014; Wang et al., 2015), and reviewed thoroughly in Boccaletti et al., 2014). We, however, are mainly interested in understanding how these layers are coordinated, and so we used a layered motif approach (Yeger-Lotem et al., 2004). Single layer motifs have been called the building blocks of networks (Milo et al., 2002), and represent distinct combinations of interactions between 2, 3 or more individuals. Our dual layered motif analysis can therefore be thought of as the dynamics of smaller groups that tile to create the group structure, or alternatively they provide information on what the average child would do in a given local social environment. It is worth noting that different combinations of motifs could give similar-looking networks, in which case the underlying mechanisms of networks formation in different classrooms may vary.

While the ordering of the layers (e.g., Aggression/Submission vs. Submission/Aggression) will determine the ID numbers of regulated motifs, the underlying data distribution is not affected, and the motif IDs can be interchanged with a look up table. Again we use *Z*-scores (with the null indicating a lack of coordination between layers) to understand the up and down regulation that characterizes relationships between two types of interactions, further elucidating the underlying interaction patterns and group dynamics in a broad manner.

We examine the 2 layer motifs of 3 types of reciprocal interactions to quantify the network structure and determine whether transitivity (not necessarily hierarchy), is the main mechanism behind their formation. With this approach we therefore test the overarching hypothesis of whether there is regulation of layered motifs. Specifically, if transitivity is the driving force in network formation, we expect up regulation of motifs that do not violate the transitive property, and down regulation of motifs that do.

## Materials and methods

The dataset is described elsewhere in detail (Boyce et al., 2012). Briefly, 338 kindergarteners (representing ~60% of the enrolled students) within 29 classrooms were sampled from 6 public school classrooms across Berkeley, California. The children were aged 4.8–6.3 years old and included 163 girls and 175 boys. The dataset was acquired from 2003 to 2005. Interactions were observed by trained research assistants through focal sampling over several weeks. Interactions scored were grouped into six overarching categories:

- [*Aggressive*] Chase, Physical Aggression, Approach, Relationship Aggression, Tease, Threat, and Verbal Aggression.
- [*Submissive*] Apologize, Compliance, Seeks Help, Retreat, and Submission.
- [*Leadership*] Directs Behavior, Reprimands, And Leadership Other.
- [*Followship*] Follow/Copy, Solicit Instruction, Followship Other, and Watching.
- [*Resource Struggle*] Object Struggle, Position Struggle, Student Attention, Teacher Attention, and Resource Struggle.
- [*Prosocial*] Offers a Gift, Offers Help, Protects, Speaks Nicely, and Prosocial Other.

All analyses were coded in Matlab [MathWorks]. For transition matrices, interactions were imported and sorted temporally by child. Both subsequent-interaction transition probabilities and probabilities of outgoing-after-incoming were normalized by child, then averaged over all children and normalized again. For the analysis of child interactions, individual transition probabilities (subsequent behavioral outputs) were used to perform a principal component analysis. The first two components were kept, all others were not plotted as each explained less than 7.5% of the variance.

For network measurements (assortativity coefficient, betweenness centrality, clustering, and efficiency) the brain connectivity toolbox was used, and we extended it for the layered network motif analysis (Rubinov and Sporns, 2010). For betweenness centrality, clustering, and local efficiency, individual values were calculated for each child as well as for individual classrooms. For assortativity, only classroom level values were calculated. Raw scores (*x*) were normalized to *Z*-scores based on random expectation:

$$\begin{array}{l} Z=\frac{x-\mu_{random}}{\sigma_{random}} \end{array}$$

Where μ is the mean and σ is the standard deviation. For each *Z*-Score, 10,000 random networks were used, constrained to have the same in- and out- degree distribution.

Layered network motif analysis similarly used *Z*-Score normalization. Each network's motif count was normalized by permuting the order of children in one network while keeping the other constant. In this way, the number of motifs remained constant within each layer of the network, but any correlation between them was destroyed:

$$\begin{array}{l} Z=\frac{x-\mu_{uncorrelated}}{\sigma_{uncorrelated}} \end{array}$$

For each *Z*-Score, 10,000 uncorrelated networks were used. *Z*-Scores were tested with a modified sign test against the null (no regulation—0). Probabilities were calculated as follows; if the majority of *Z*-scores (*z*+) for a specific motif were positive the *p*-value was calculated as the chance of observing this result out of the total number of classrooms (classes); p=(classesz+)0.5classes. Similarly if there was a majority of negative *Z*-scores (*z*−), p=(classesz-)0.5classes otherwise the probability was set at 1 if the majority of *Z*-scores were not regulated (i.e., 0). The *p*-value was evaluated at an alpha value of 0.05 divided by the number of possible layered-network motifs—for dyadic motifs (9 possible motifs) this value is 0.0056, for triadic motifs (710 possible motifs) this value is 7.0423 × 10^−5^.

## Results

**Figure 1:**
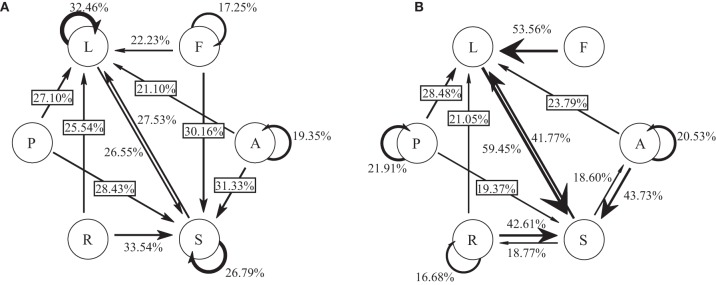
**Transition probabilities between interaction types. (A)** The transition probabilities between subsequent outgoing interaction types. **(B)** The transition probabilities of a subsequent outgoing interaction given a specific incoming interaction type. Interaction types: (L)eadership, (F)ollowship, (A)ggression, (S)ubmissive, (R)esource struggle, and (P)rosoci.

To visualize the connectivity and temporal order of interactions across all classrooms, we used transition matrices indicating the percent likelihood that one behavioral output will follow another (e.g., there is a 22.23% probability of leading after following; Figure 1A), and the likelihood of a behavioral output given a specific behavioral input (e.g., leadership is the most likely response (28.48%) to a prosocial interaction; Figure 1B). Using principal component analyses on these transition matrices we can see slight group separations based on interaction patterns (Figure S1). We found a small group of kindergarteners that seemed separated from the rest of their classmates based on their likelihood to move from a prosocial interaction to a leadership interaction, lending support to the idea that while an average kindergartener may be a useful model, a child's behavioral repertoire can be a diagnostic tool to categorize children.

When moving from the individual child as a focus to a more group-level analysis, we aim to quantify the organization that classrooms exhibit. Social interaction networks can be described with four main parameters:

- *Betweenness centrality*: The number of shortest paths (between other nodes) that traverse a given node (e.g., the importance of an average node for information flow and network cohesion).
- *Clustering coefficient*: The likelihood that node neighbors interact amongst themselves (e.g., in a friendship network, are a node's friends are friends).
- *Assortativity*: The correlation between a node's degree (number of incoming and outgoing interactions) and the degree of his neighbors (e.g., do popular kids interact more with other popular kids).
- *Efficiency*: The inverse of the average shortest paths through a network (e.g., how quickly could information flow through the network).

The calculation of these measures are described in detail elsewhere (Newman, 2010). Once networks were extracted from each classroom for each interaction type, we compared the *Z*-scores for each network parameter among all students and classrooms to look at relationships between underlying mechanisms or characteristics in the formation of different interaction networks. *Z*-scores allow us to determine whether or not the classroom is up or down regulating aspects of organization above/below what is expected randomly. The highest correlation was found for the clustering coefficient of submissive and leadership interactions for all classrooms (*r*^2^ = 0.58242; Figure S2). The assortativity (*r*^2^ = 0.21406) and efficiency (*r*^2^ = 0.17387) measures for these social interactions were also correlated, but the betweenness centrality measure was not (*r*^2^ = 3.519e-05). This is a preliminary analysis; we look at correlations between all interaction types for both the same and different network measures at both the classroom and student levels. There is little to no statistical rigor associated with these comparisons, however, they provide preliminary data for future hypotheses.

**Figure 2:**
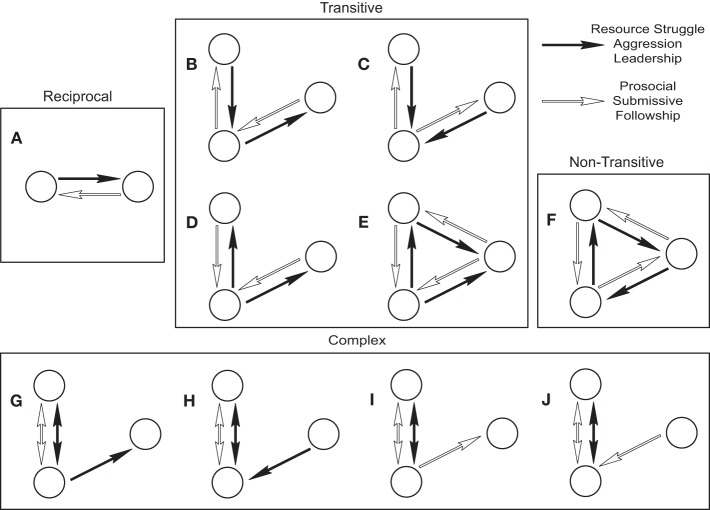
**Reciprocal, transitive, intransitive, and complex relations between interaction types. (A)** Illustration of dyadic reciprocation **(B)** Pass along **(C)** Focused A **(D)** Focused B **(E)** Complete. **(F)** The non-transitive relationship **(G–J)** Complex relationships that are significantly over-represented in both Aggressive/Submissive and Leadership/Followship networks.

To examine patterns in the reciprocal interaction pairings we looked at dyadic and triadic motifs (2 and 3 individuals respectively) of all types, rather than simply the transitive and non-transitive (cyclic) motifs. We found that the interaction pairings were not simply reciprocated in the dyadic motifs of Aggression/Submissive, Leadership/Followship and Resource Struggle/Prosocial networks (reciprocated interactions accounting for 46.78, 45.67, and 16.46% of all dyadic interactions respectively) (Figure 2A; see Table S1). To examine the level of similarity between motifs formed in the different layered networks we looked at the correlations between all up/down regulated motifs between each layered network. Down-regulated motifs were highly correlated among all three layered networks, however, the up-regulated ones were only correlated among Aggressive/Submissive and Leadership/Followship networks (Figure S3). These universally down-regulated motifs are often patterns where only one interaction type is present, indicating that the relationship between our interaction pairs is non-trivial and suggesting that their regulation is interdependent.

We found up regulation of one transitive triadic motif (termed “Focused A”) in the Aggressive/Submissive and Leadership/Followship networks (Figure 2C and Table 1). Due to our relatively strict *p*-value correction, other transitive triadic motifs (“Pass Along,” “Focused B,” and “Transitive Triangle”; Figures 2B,D,E) for Aggressive/Submissive and Leadership/Followship networks had positive *Z*-scores and relatively small *p*-values but failed to achieve statistical significance. We found no evidence of down regulation of the non-transitive cyclic motif in the Aggressive/Submissive and Leadership/Followship networks (Figure 2F). In addition to these motifs commonly discussed in relation to hierarchy, we found up regulation of 4 complex triadic motifs (Figures 2G–J; Table 1). We note that the complex motifs of Figures 2G,H are the reciprocals of Figures 2I,J, and together with the high correlation in Figure S3, indicate that the relationship of Leading to Following is more similar to the relationship of Submissive to Aggressive than Aggressive to Submissive. We found an additional 27, 185, and 180 up/down regulated motifs (for Resource/Prosocial, Aggression/Submission and Leadership/Followship respectively; Table S2). Taken together, the results suggest transitivity is not the only social relationship organizing these interactions.

**Table 1:** **Observed, expected and ***Z***-scores for motifs displayed in Figure 2**.

	Motif # (^*^)	**Figure 3**	**Frequency**		**Expected Frequency**		***Z*****-Score**		***P*-value**
			**Mean**	**Std**	**Mean**	**Std**	**Mean**	**Std**	
Resource struggle/Prosocial	3 (3)	A	2.93	2.62	1.78	1.16	0.94	1.60	1.87E−02
	470 (470)	B	0.45	0.91	0.10	0.09	1.13	2.96	7.99E−03
	424 (574)	C	0.17	0.60	0.07	0.07	0.44	2.10	8.85E−04
	574 (424)	D	0.41	1.15	0.07	0.08	1.11	2.92	1.87E−02
	622 (622)	E	0.00	0.00	0.00	0.00	−0.03	0.04	1.00E+00
	678 (678)	F	0.00	0.00	0.00	0.00	−0.01	0.02	1.00E+00
	44 (242)	G	0.45	0.99	0.29	0.49	0.22	0.99	1.00E+00
	45 (243)	H	0.41	0.82	0.25	0.42	0.28	1.12	1.00E+00
	242 (44)	I	1.66	3.56	0.69	1.17	0.65	1.99	1.00E+00
	243 (45)	J	1.55	3.70	0.64	1.10	0.35	1.08	1.00E+00
Aggressive/Submissive	3 (3)	A	13.21	5.45	5.47	2.12	3.73	1.75	**1.86E**-**09**
	470 (470)	B	3.24	3.39	0.44	0.23	3.69	4.14	7.99E−03
	424 (574)	C	4.00	3.55	0.47	0.30	3.99	3.22	**4.42E**−**05**
	574 (424)	D	2.62	2.91	0.27	0.19	4.23	4.70	8.85E−04
	622 (622)	E	0.66	1.20	0.05	0.05	2.57	4.21	9.67E−02
	678 (678)	F	0.10	0.41	0.01	0.01	0.81	3.28	1.00E+00
	44 (242)	G	8.52	8.14	2.24	2.03	2.67	1.84	**6.81E**-**06**
	45 (243)	H	9.10	7.38	2.63	2.02	2.48	1.75	**7.56E**−**07**
	242 (44)	I	1.86	1.94	1.29	1.17	0.46	1.15	9.67E−02
	243 (45)	J	1.79	1.72	1.02	0.91	0.69	1.14	6.44E−02
Leadership/Followship	3 (3)	A	16.66	10.25	5.93	2.92	4.62	2.31	**1.86E**−**09**
	470 (470)	B	6.21	8.79	0.47	0.37	5.90	6.63	8.85E−04
	424 (574)	C	6.14	8.09	0.63	0.58	4.79	4.88	**7.56E**−**07**
	574 (424)	D	2.72	3.30	0.30	0.18	3.65	3.91	7.99E−03
	622 (622)	E	1.45	2.40	0.08	0.11	3.67	5.00	1.44E−01
	678 (678)	F	0.10	0.41	0.00	0.01	0.82	3.27	1.00E+00
	44 (242)	G	0.90	1.35	0.69	0.65	0.14	0.98	1.87E−02
	45 (243)	H	1.55	1.92	0.85	0.74	0.52	1.18	1.00E+00
	242 (44)	I	8.14	7.80	1.65	1.37	3.03	2.46	**4.42E**−**05**
	243 (45)	J	5.00	4.52	1.12	0.97	2.37	1.75	**4.42E**−**05**

*Significant Z-Scores are indicated in bold*.

* *Motifs IDs are indicated along with their reciprocal IDs if the layers were reversed*.

## Discussion

When examining dominant and subordinate behaviors within a population, the ways in which these behaviors or interactions are measured can influence the results identified. There are multiple methods of establishing winner/loser identity based on a particular signal, or behavior following a fight. Oftentimes pitting individuals against each other is done in round-robin or tournament set-ups to classify individuals as dominant or subordinate on the basis of their number of wins. Other times proxies of dominance, including body size (Archie et al., 2006, Fujimoto et al., 2011), age (Côté, 2000; Archie et al., 2006) or specific markings (Tibbetts and Lindsay, 2008), are used. The application of these calculated hierarchies to the group as a whole is limited, both in implied transitivity as well as in the sense that dyadic competition is likely not equivalent to combat within a group setting (Haemisch et al., 1994). This is in addition to the fact that designing tournaments that minimize previous-match effects are non-trivial (Russell, 1980). To generalize, the methods with which dominance and subordination are coded or classified dictates the scope of the results that can be appropriately interpreted. Furthermore, examining patterns of interactions with the intention of finding hierarchies limits and/or biases the extent to which the results characterize the social interaction patterns themselves. That is, searching for an answer to a specific question or phenomenon may lead to the acceptance of an incomplete explanation.

Here, we take a hierarchy-neutral approach to examine all possible underlying patterns in the formation of layered networks of “reciprocal” interactions. Like other studies specifically examining hierarchy, we examine interactions between individuals looking for transitive and non-transitive relationships that may indicate a hierarchical structure in the classrooms of kindergarten children (Shizuka and McDonald, 2015), with a couple of caveats. First, we do not focus on only a subset of the patterns, instead looking at all motifs to take a comprehensive approach to the mechanisms that could potentially be contributing to classroom structure. Second, we do not consider order in the hierarchical structure. That is, we avoid imposing the generally accepted order of aggressive or leading individuals being dominant to submissive or following individuals. We do this for two reasons. First, we did not have specific questions in mind where such an ordering was required and second, the order applied to these interaction types depends on the frame of reference, or more specifically, what would be defined as a “win.” Specifically, if we were to define winning as controlling information in a group, all six interactions we examine could be considered “dominant” to their partner interaction depending on the frame of reference. Leaders could be dominant because they choose which followers to share information with or followers could be dominant because they get information from all those they follow; aggressors fight and access information, submissives do not have to fight to get information; prosocial individuals control sharing of information or those involved in resource struggle retain information they hold. Because we do not impose order on the interactions we are investigating, we need not refer to one or the other as dominant or subordinate (those labels would depend on context), rather we simply refer to their networks as having a transitive structure or not.

We first showed that these interactions were not as reciprocal as they are often considered (Figures 1B, 2). That is, a Leadership interaction does not ensure a Followship response, similarly for Aggression and Submission, and Prosocial and Resource Struggle. We note here that these dyadic motifs do not encompass order of interactions or timing as the individual analysis of transitions do. Both incoming Aggression then outputting Submission and incoming Submission then outgoing Aggression, over any time period, would be considered reciprocal. When we examine the regulated motifs in all three layered networks, we find that there are similarly high correlations in down-regulated motifs between the three layered networks (Figure S3A). However, only the Aggressive/Submissive and Leadership/Followship layered networks showed correlation for the positively regulated motifs (Figure S3B). This suggests that while there is a shared structure of highly unlikely motifs (mostly consisting of motifs of one layer only), the Resource Struggle/Prosocial has a different structure compared to the Leadership/Followship and Aggressive/Submissive networks.

Figure 2 shows the up regulated motifs common to Aggression/Submission and Leadership/Followship layered networks. The most intuitive are those in Figures 2B–E, and we can easily imagine kindergarten children behaving in such reciprocal ways (e.g., two kindergarteners being mean to one other; Focused A). The final 4 motifs shown in Figure 2 are more difficult to understand in terms of children's interactions. They illustrate how non-intuitive, abstract, and non-hierarchical patterns of interactions can be found using unbiased network and motif approaches. Furthermore, by examining the order of layers in the networks, we can observe which behavioral relationships are similar. We see higher correlations between Leadership/Followship and Submissive/Aggressive than Leadership/Followship and Aggressive/Submissive (Figures 2G–J and Figure S3). This hints that the mechanisms and environmental pressures which shape the interactions of social “leaders” may be more similar to the interactions of social “submissives” and not social “aggressives.”

The up regulation of the transitive motif Focused A indicates that transitivity is at least partially involved in the formation of these layered network structures. Transitivity is perhaps the most important property of structural hierarchy in a group (the other two being stability and linearity) as it, by definition (A>B and B>C then A>C), creates the characteristic orderly stratified layers. We also found complex combinations of the expected reciprocal interactions: two individuals in a fully connected dyad interacting with a third (Figures 2G–J). None of these can be created by overlapping the above transitive motifs, and their up regulation in both Aggressive/Submissive and Leadership/Followship networks is intriguing. While we cannot rule out “un-resolved” hierarchy (i.e., these are fingerprints of hierarchical establishment itself), another exciting possibility hints at more underlying mechanisms of network structure than simply hierarchy.

Boyce examined the influence of socioeconomic status on hierarchies in the same kindergarten classroom data we have presented here (Boyce et al., 2012). While we were aware that classrooms were not homogeneous for characteristics including SES, teacher profiles, instructional methods etc., we did not separate the dataset according to these external factors. That is, we did not perform separate analyses on low SES vs. high SES, similar for other potentially confounding factors which may be at play. We note, however, that our statistic (sign test) is non-parametric and does not require all classrooms to behave similarly to draw overall conclusions.

In children, the ability to examine behaviors in both one-on-one situations and within a larger social group could be used as a tool for finding behavioral abnormalities. Much of the diagnosis process for autism is based on behavioral observations of a child and understanding how such an individual would appear within, be affected by, and affect their social environment, could go a long way to clearer diagnoses (Stone et al., 1990). A potential avenue of diagnostic implication is the nurturing of extended social interactions within a group. By identifying normal rates of participation within a group via network motifs, one may be able to identify abnormal motif participation by students who may require more intervention to be able to properly flourish within a highly structured group (so called “orchid” as opposed to “dandelion” children Ellis and Boyce, 2008). The methodology outlined here could therefore provide a framework for identifying children to more readily ensure their support in an appropriately protective environment if they participate in abnormal network relationships (i.e., motifs).

The mechanisms used to understand social networks of kindergarten children here are also applicable on a more general scale to any group of individuals where interactions and identities can be readily observed and scored. We have shown that social interactions are not isolated behaviors and that the commonly paired types (Leadership/Followship, Aggression/Submission) interact with each other in a complex manner unpredictable given simple rules of reciprocation and transitivity. We further predict this method can be extended to more than two interaction types and to quadratic motifs and as computational resources improve, and will continue to improve our understanding of the multi-layered networks that more accurately represent the natural social environment in humans and other animals.

## Author contributions

JL conceived of study together with JS and MG. Contributed to writing the paper with JS and MG and co-authors. JS wrote the code, conceived study and helped write paper. MG wrote first draft, conceived study. WB, NB, and NA provided data for analysis.

## Funding

This work was funded grants awarded to JDL by CIHR, NSERC, CRC, and CIFAR.

### Conflict of interest statement

The authors declare that the research was conducted in the absence of any commercial or financial relationships that could be construed as a potential conflict of interest.

## Supplementary material

Figure S1 **Principal Component (PC) analysis of subsequent interaction transitions**. The first and second principal components account for 22.11% of the total variance in a kindergartener's transition probabilities between interaction types. There appears to be a subset of kindergarteners that are defined by their shared propensity to transition from Prosocial to Submissive. Factors are plotted if their loading was greater than 0.25. Kindergarteners are colored by classroom.

Figure S2 **Correlation analysis between clustering of Submissive interactions and Leadership interactions. (A)** The correlation when measuring local clustering. **(B)** The correlation when measuring the average clustering per classroom. Kindergarteners are colored by classroom.

Figure S3 **Correlation analysis using the ***Z***-scores of the triadic motifs. (A)** The Leadership/Followship network displays correlated significant motifs when compared to the Submissive/Aggressive network, even with very highly up-regulated motifs. **(B,C)** The Resource Struggle/Prosocial network correlates well with under-represented motifs, but poorly with over represented motifs with both the Submissive/Aggressive and Leadership/Followship networks. Motifs were excluded if they were never detected as significantly different (*Z*-scores) in any network. The ordering of the network layers was arranged to generate the higher correlation.

Table S1 **Observed, expected and ***Z***-scores for dyadic motifs**. Significant *Z*-Scores are indicated in bold. ^*^Motifs IDs are indicated along with their reciprocal IDs if the layers were reversed.

Table S2 **Observed, expected and ***Z***-scores for triadic motifs**. Significant *Z*-Scores are indicated in bold. ^*^Motifs IDs are indicated along with their reciprocal IDs if the layers were reversed.
