# Supplementary figures and images for: Layered Social Network Analysis Reveals Complex Relationships in Kindergarteners
Source: Front Psychol. 2016 Mar 2;7:276. doi: 10.3389/fpsyg.2016.00276 (PMC4773607; doi:10.3389/fpsyg.2016.00276)

Figure S1

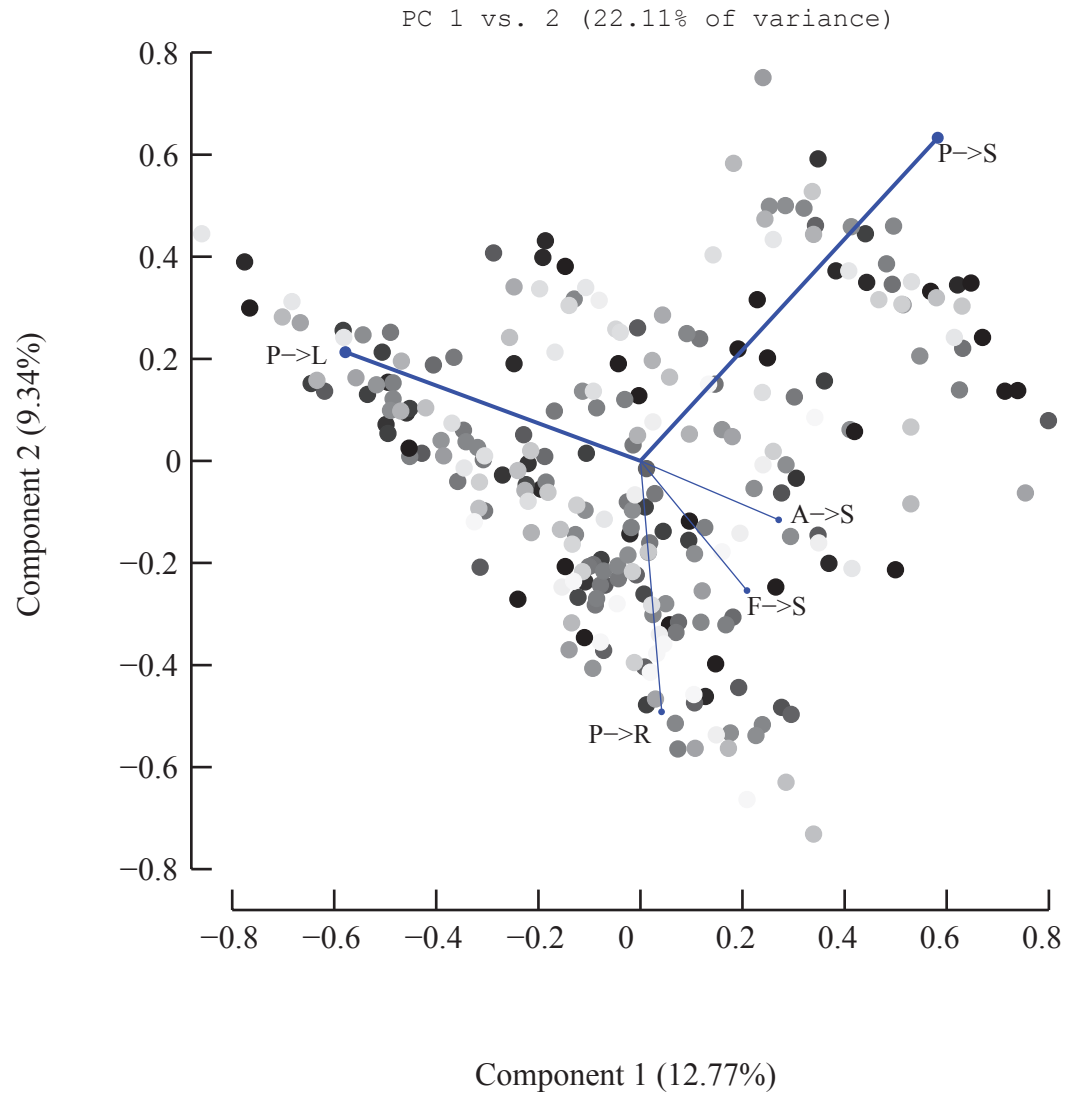

Supplement: Figure S1 — Principal Component (PC) analysis of subsequent interaction transitions. The first and second principal components account for 22.11% of the total variance in a kindergartener's transition probabilities between interaction types. There appears to be a subset of kindergarteners that are defined by their shared propensity to transition from Prosocial to Submissive. Factors are plotted if their loading was greater than 0.25. Kindergarteners are colored by classroom. [file Image1.PDF]

Figure S2

A

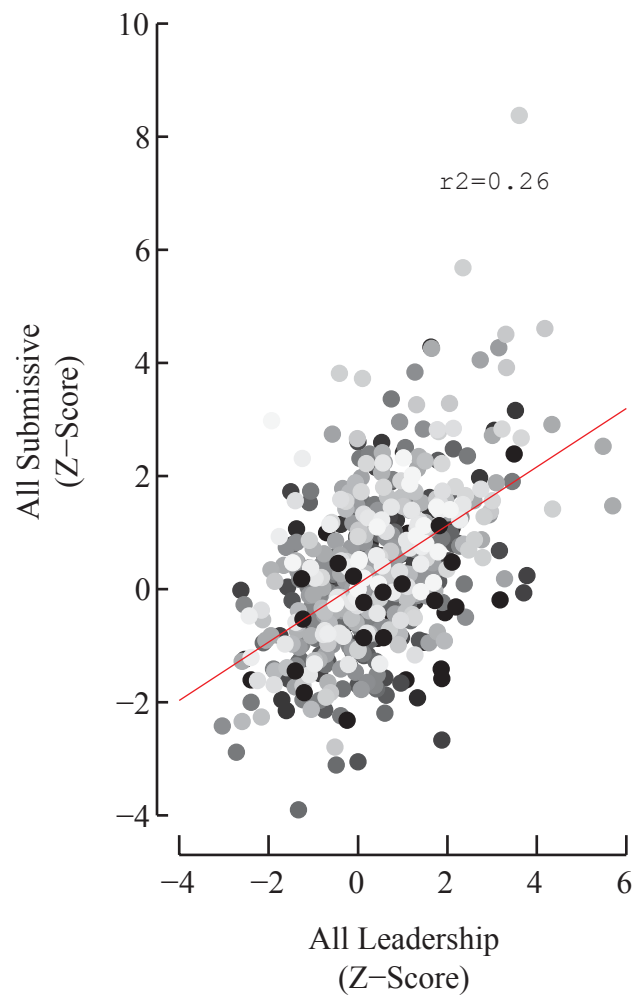

B

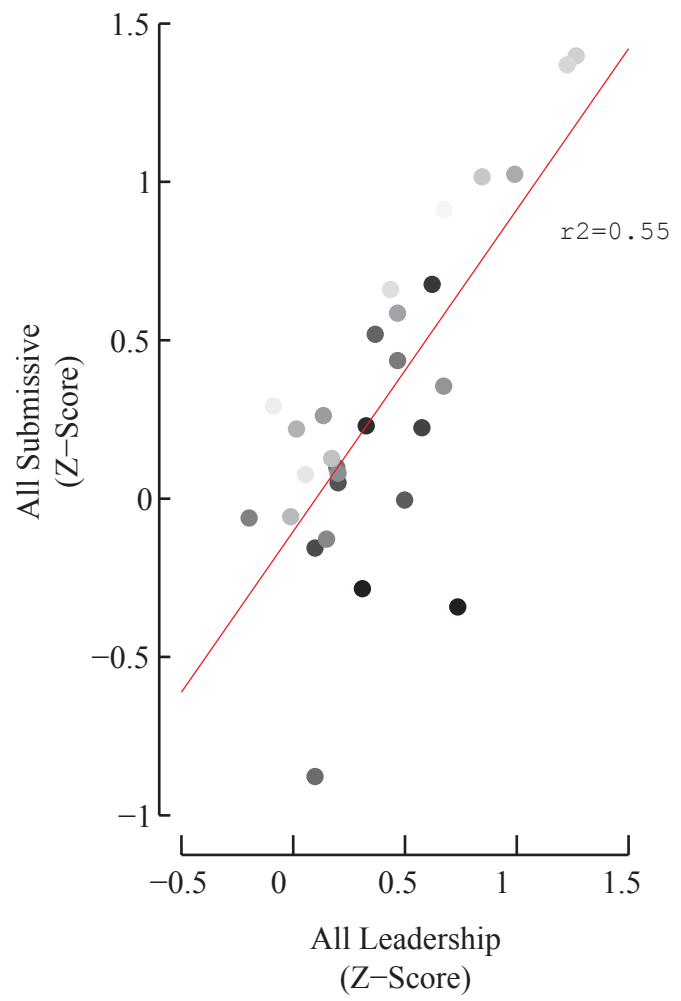

Supplement: Figure S2 — Correlation analysis between clustering of Submissive interactions and Leadership interactions. (A) The correlation when measuring local clustering. (B) The correlation when measuring the average clustering per classroom. Kindergarteners are colored by classroom. [file Image2.PDF]

Figure S3

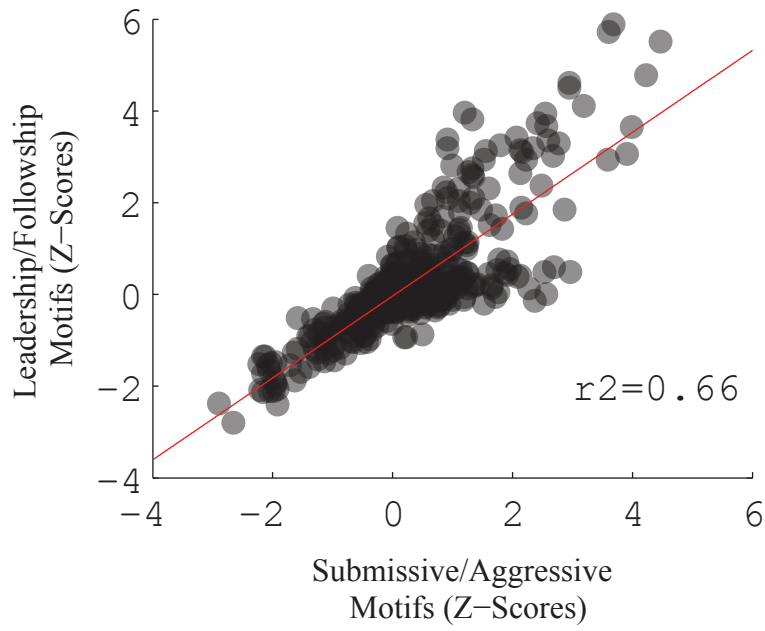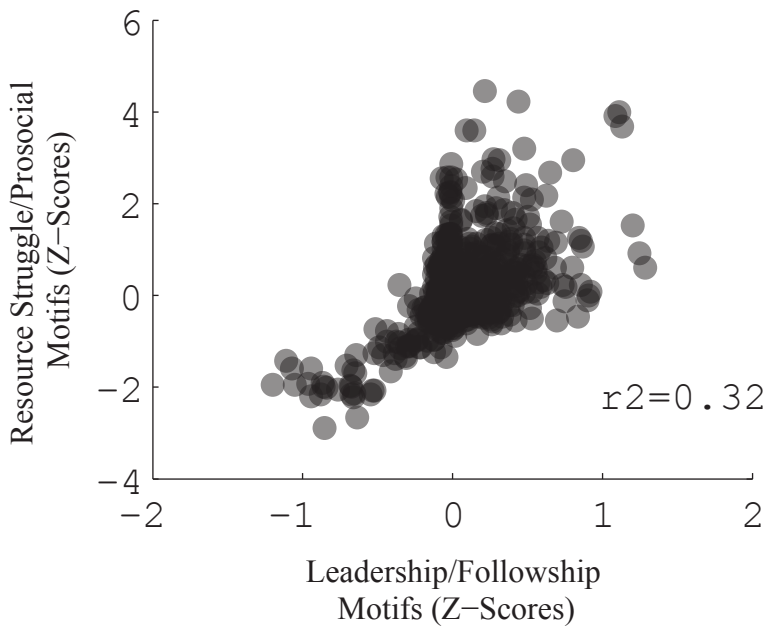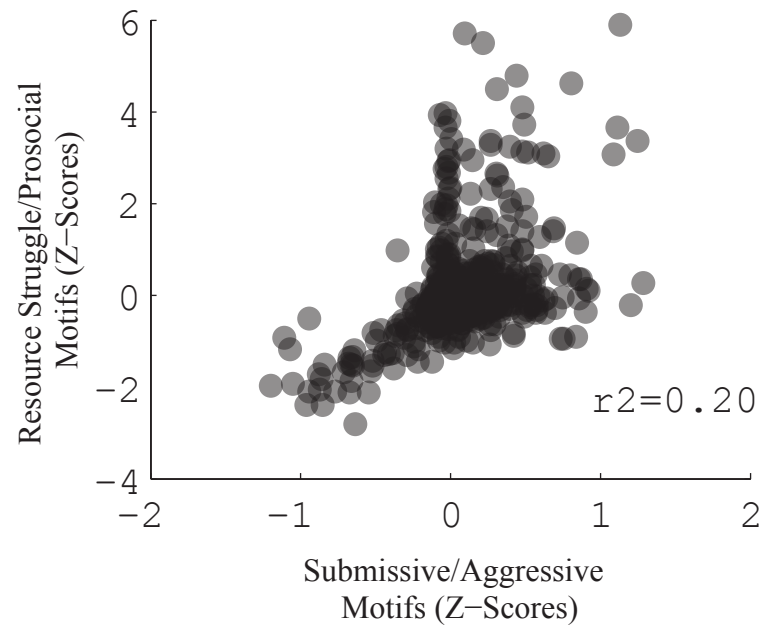

Supplement: Figure S3 — Correlation analysis using the Z-scores of the triadic motifs. (A) The Leadership/Followship network displays correlated significant motifs when compared to the Submissive/Aggressive network, even with very highly up-regulated motifs. (B,C) The Resource Struggle/Prosocial network correlates well with under-represented motifs, but poorly with over represented motifs with both the Submissive/Aggressive and Leadership/Followship networks. Motifs were excluded if they were never detected as significantly different (Z-scores) in any network. The ordering of the network layers was arranged to generate the higher correlation. [file Image3.PDF]
